# Prognosis does not change the landscape: palliative home care clients experience high rates of pain and nausea, regardless of prognosis

**DOI:** 10.1186/s12904-021-00851-x

**Published:** 2021-10-20

**Authors:** Nicole Williams, Kirsten Hermans, Tara Stevens, John P. Hirdes, Anja Declercq, Joachim Cohen, Dawn M. Guthrie

**Affiliations:** 1 Department of Kinesiology and Physical Education, Wilfrid Laurier University, 75 University Ave W, Waterloo, ON Canada; 2 End-of-life Care Research Group, University of Brussels (VUB) and Ghent University (UGent), Laarbeeklaan 103, 1090 Brussels, Belgium; 3 University of Leuven (KU Leuven), LUCAS, Minderbroedersstraat 8 box 5310, 3000 Leuven, Belgium; 4 School of Public Health and Health Systems, University of Waterloo, 200 University Ave W, Waterloo, ON Canada; 5 University of Leuven (KU Leuven), CESO, Minderbroedersstraat 8 box 5310, 3000 Leuven, Belgium; 6 Department of Health Sciences, Wilfrid Laurier University, 75 University Ave W, Waterloo, ON Canada

**Keywords:** Palliative care, interRAI, Home care, Prognosis

## Abstract

**Background:**

Most individuals who typically receive palliative care (PC) tend to have cancer and a relatively short prognosis (< 6 months). People with other life-limiting illnesses can also benefit from a palliative care approach. However, little is known about those who receive palliative home care in Ontario, Canada’s largest province. To address this gap, the goal of this project was to understand the needs, symptoms and potential differences between those with a shorter (< 6 months) and longer prognosis (6+ months) for individuals receiving PC in the community.

**Methods:**

A cross-sectional analysis was conducted using interRAI Palliative Care (interRAI PC) assessment data collected between 2011 and 2018. Individuals with a shorter prognosis (< 6 months; *n* = 48,019 or 64.1%) were compared to those with a longer prognosis (6+ months; *n* = 26,945) across several clinical symptoms. The standardized difference (stdiff), between proportions, was calculated to identify statistically meaningful differences between those with a shorter and longer prognosis. Values of the stdiff of 0.2 or higher (absolute value) indicated a statistically significant difference.

**Results:**

Overall, cancer was the most prevalent diagnosis (83.2%). Those with a shorter prognosis were significantly more likely to experience fatigue (75.3% vs. 59.5%; stdiff = 0.34) and shortness of breath at rest (22.1% vs. 13.4%; stdiff = 0.23). However, the two groups were similar in terms of severe pain (73.5% vs. 66.5%; stdiff = − 0.15), depressive symptoms (13.2% vs. 10.7%; stdiff = 0.08) and nausea (35.7% vs. 29.4%; stdiff = 0.13).

**Conclusions:**

These results highlight the importance of earlier identification of individuals who could benefit from a palliative approach to their care as individuals with a longer prognosis also experience high rates of symptoms such as pain and nausea. Providing PC earlier in the illness trajectory has the potential to improve an individual’s overall quality of life throughout the duration of their illness.

**Supplementary Information:**

The online version contains supplementary material available at 10.1186/s12904-021-00851-x.

## Background

As the population continues to age and people continue to live longer, there is a growing number of individuals living with chronic illnesses such as Alzheimer’s dementia, organ failure, stroke and chronic lung diseases. Within Canada, roughly three in ten individuals (32%) have a chronic illness, with 70% of all deaths occurring from a chronic disease [1]. Many organizations that provide care to these individuals agree that it is important to integrate a palliative care (PC) approach as early as possible for those with a life-limiting illness [2]. PC that is provided earlier in the illness trajectory has the potential to improve overall quality of life, reduce symptom burden, fulfill goals and wishes of the dying individual and may lead to reduced health care service use [3, 4].

In 2008, it was estimated that only 16 to 30% of individuals had access to formal palliative care services [5], the majority of whom had a cancer diagnosis [6]. In 2011, in Ontario, 80 stakeholders came together to develop a declaration of understanding, which outlined a new vision and plan for how PC should be delivered in the province. In this plan, it was stated that the availability of PC should not be restricted to individuals in the final weeks of life with a cancer diagnosis. Instead, PC would ideally be available for all adults and children with a progressive life-limiting illness and offers support to the patient and their family throughout the entire spectrum of care. Additionally, anyone who wishes to remain at home should be able to access the care they need through primary care providers within the community [6].

Even though there is strong evidence that providing a palliative approach to care that starts early in the illness trajectory is beneficial, the literature still shows that the majority of care is focused on the last weeks or days of life [4, 7]. There are a number of obstacles that may prevent a timely approach to care, including the lack of standardized criteria to determine eligibility. The trajectory of chronic life-limiting illnesses are often unpredictable and can therefore make it difficult to identify the most appropriate time to initiate PC [2, 8]. As a result, clinicians have often relied on prognosis (e.g., death foreseeable in short term) rather than on palliative care needs. The use of the “surprise question” (i.e., “Would you be surprised if the person died in the next 12 months?”) [9] alongside other palliative care needs assessment tools (i.e., Palliative Performance Scale) [8, 10] have been proposed as a means to identify eligibility to receive PC in an earlier stage. In practice, their application in standard care has been rather limited.

For the most part, older adults prefer to “age in place” and remain in their own homes for as long as possible [11]. This is also true as it relates to the place of death and where the vast majority of individuals would prefer to receive PC [1, 12]. Even though the majority of Canadians express a wish to receive palliative care at home, there is little information within the literature around the symptom burden and care needs of these individuals. A recent population-level retrospective cohort study for all decedents in Ontario between 2010 and 2012 found that PC services are more infrequently delivered in community settings, especially for those with other life-limiting illness, besides cancer [13]. In 2017, all provinces and territories in Canada agreed to a common statement of principles on shared health priorities, highlighting the need to improve access to home and community care, including palliative home care [14]. In order for health care professionals and policy makers to provide quality palliative care in the home environment, more information is needed on exactly who is receiving PC in Ontario, Canada’s largest province.

Currently, the interRAI Palliative Care (interRAI PC) assessment is only used in Ontario to assess individuals receiving PC in the community. The interRAI PC was developed in 2003 by interRAI, a non-profit organization of roughly 100 clinicians and researchers representing 35 countries who develop and test standardized assessments for use in various populations, including palliative care. The interRAI PC offers a wealth of data at the person-level and offers insights in the needs of individuals receiving PC in the community. The current body of literature has limited information on who is receiving PC in the community in Ontario. To address this gap using existing interRAI PC data, the current study aims to understand the needs, symptoms and potential differences in characteristics for those with both a shorter and longer prognosis for individuals receiving palliative care in the community.

## Methods

### Study design

This cross-sectional study utilized secondary data collected using the interRAI Palliative Care (interRAI PC) assessment in Ontario. The interRAI PC is a comprehensive assessment instrument that identifies person-specific palliative care preferences, symptoms and needs to support health professionals in the care planning process [15, 16]. The assessment covers key domains such as prognosis, cognitive and functional status, communication, mood, psychosocial well-being and spirituality. A number of studies support the validity and reliability of the interRAI assessments [17, 18]. Assessments are completed in the individual’s home by trained assessors (e.g., registered nurses and other allied health professionals) in the form of a semi-structured interview. Answers on the interRAI PC are based on information from direct observations with the individual, discussions with informal caregivers, and other health professionals (e.g., primary care physician), and review of available medical records, as needed.

### Sample

All interRAI PC clients who had a completed assessment between 2011 and 2018 were included, representing the most recent information available for Ontario. For individuals with multiple assessments completed within this time frame, the most recent assessment was retained for analysis. This resulted in a total sample of 74,964 unique individuals. This sample represents a mix of individuals that have been on service for a longer period of time (e.g., had more than one assessment; 24.8% of the sample), as well as those that would have recently started service (75.2% of sample). We ultimately decided to utilize each individual’s most recent assessment for analysis in order to take a cross section of all clients on service that is as close to the current landscape as possible. The data were de-identified before being shared with the research team. Prognosis was captured with a single item on the interRAI PC with four response options including death imminent (within days), less than six weeks, six weeks or longer, but less than six months and six months or longer to live. To differentiate those with a short versus long prognosis, we decided to collapse and dichotomize the prognosis variable comparing those with less than six months to live (*n* = 48,019; 64% of the sample) to those with more than six months to live (*n* = 26,945; 36%). Eligibility for PC has often been determined by proximity to death and tends to fall within the last weeks or months of life [5]. This is also consistent with previous research that has used the surprise question: “would you be surprised if this patient were to die within 6-12 months?” to determine initiation of palliative care [19]. The project was reviewed and approved by the Research Ethics Board at Wilfrid Laurier University (REB #5844).

### Measures

There are five health index scales embedded within the interRAI PC assessment which are automatically generated upon completion of the assessment:

1. *The Cognitive Performance Scale (CPS)* is scored from zero to six and includes four items, namely, short-term memory, cognitive skills for daily decision making, expressive communication and independence in eating. A cut-point of two or higher was used to indicate at least moderate impairment in cognitive functioning. The scale has been validated against the Mini Mental State Exam (MMSE) [20] and is correlated with the Montreal Cognitive Assessment (MoCA) [21].
2. *The Pain Scale* includes two items which capture both the frequency and intensity of pain. The scale is scored from zero (no pain) to three (severe/daily pain) and has been validated against the Visual Analog Scale [22]. A score of two or higher was used to indicate severe/daily pain, since research has shown an important increase in the VAS score among those with a score of 2 or higher [22].
3. *The Activities of Daily Living Self-Performance Hierarchy Scale (ADL-H)* is scored from zero (independent) to six (total dependence) for items including bathing and dressing. In line with previous research, a cut-point of two or higher indicated at least moderate difficulty completing ADL tasks independently [23, 24]. The ADL-H is a valid and reliable measure of functional ability [25].
4. *The Depression Rating Scale (DRS)* is scored from zero to fourteen and includes items pertaining to both mood and behavior. A score of three or higher has been found to be predictive of a clinical diagnosis of depression [26].
5. *The Pressure Ulcer Risk Scale (PURS)* is scored from zero to eight and includes items such as impaired bed mobility, bowel incontinence, weight loss and history of a resolved pressure ulcer [27]. In line with previous research, a cut-point of three or higher was used to indicate those at moderate risk of developing a pressure ulcer [28].

### Other measures

Several other dichotomous variables (yes/no) around physical, psychosocial and spiritual characteristics were examined including a recent fall in the last 90 days, difficulty falling asleep/staying asleep, too much sleep, shortness of breath, fatigue, nausea, vomiting, fluctuating state of consciousness, acute change in mental status, hospital and emergency department visits, a wish to die now, being at peace with life, finding guidance in religion or spirituality, being accepting of their situation as well as having a sense of completion on transfer of financial, legal and other formal responsibilities.

We also explored diagnosis, which is collected on the interRAI PC in a free-text format. A detailed description on how free-text entries were recoded into 11 diagnostic groups can be found elsewhere [29]. In summary, the free-text data were coded into categorical variables based on the *International Classification of Diseases, Tenth Revision (ICD-10)* categories. Since it was common for individuals to have multiple diagnoses, the diagnostic groups are not mutually exclusive. The 11 diagnostic groups included cancer, circulatory diseases, respiratory diseases, nervous/mental disorders, digestive disorders, metabolic/endocrine diseases, musculoskeletal diseases, as well as diseases of the skin, eye, ear and congenital diseases. We also explored the number of comorbid chronic conditions present (0-2 vs. 3+). Finally, in terms of caregiver characteristics, we examined the Caregiver Risk Evaluation (CaRE) algorithm, which is a decision-support tool that is used to assess the risk of caregiver burden, ranging from low risk to very high risk [30].

### Clinical assessment protocols (CAPs)

Within the interRAI PC, eight Clinical Assessment Protocols (CAPs) can be generated from data elements found in the assessment. CAPs are used to assist care coordinators who complete the interRAI PC with care planning and can also highlight areas of need that may benefit from treatment, additional assessment or referral [16]. The eight CAPs that can be generated include the following domains: delirium, dyspnea, fatigue, mood, nutrition, pain, pressure ulcers and sleep disturbance. Each CAP stratifies individuals into distinct triggering categories based on their level of difficulty with the problem. Individual CAP triggering is unique to each CAP, and the language used to determine who triggers each CAP is slightly different. For example, the delirium CAP has two triggering levels (not triggered/triggered), while the mood CAP has three triggering levels (not triggered/triggered with single mood symptom/triggered with multiple mood symptoms; Additional Table 1). Details on the full CAP development can be found elsewhere [31]. Since we were interested in comparing those with a shorter vs. longer prognosis, and not the individual triggering rates, we decided to collapse the CAP triggering categories to triggered (yes/no). Additional Table 2 includes a full breakdown of the CAP triggering rates for the overall sample.

There was some degree of missing data in seven of the eight CAPs, however the number of missing data is due to how the assessment is completed. For example, the delirium, fatigue, sleep disturbance and mood CAPs are not calculated for individuals who are considered comatose (missing = 636). For the pressure ulcer and nutrition CAP, some of the specific items required to populate the CAP were missing. Case managers in Ontario have the option of using an abbreviated version of the interRAI PC assessment, based on their clinical judgement (i.e., do they feel the full interRAI PC is warranted or not for this individual). This shorter version does not include the comprehensive set of items, resulting in an inability to calculate some of the CAPs.

### Analysis

Demographic and clinical characteristics between individuals with a prognosis of at least six months to live compared to those with less than six months to live were analyzed using the chi-square statistic. Given the large sample size and potential for type I error, we used an absolute standardized difference (stdiff; similar to a z-score) of 0.2 or higher to identify statistically meaningful differences between those with a shorter and longer prognosis. This cut-point was used to represent at least a small effect size [32]. The standardized difference is the difference in the two proportions, divided by an estimate of the prevalence of the covariate in each of the two groups [33]. Standardized differences are one metric to understand the effect size when comparing two proportions. All statistical analyses were completed using SAS, version 9.4 [34]. The study followed the REporting of Studies Conducted using Observational Routinely-Collected Data (RECORD) guidelines [35], which is an extension of the STrengthening the Reporting of OBservational studies in Epidemiology (STROBE) guidelines [36].

## Results

In the sample of 74,964 individuals, 64.1% had a prognosis of less than six months to live, which included individuals that had death listed as imminent (2.4%; *n* = 1839), six weeks or less to live (10%; *n* = 7515) and longer than six weeks to live, but less than six months (51.6%; *n* = 38,665). The remaining group (35.9%) had a prognosis of six months or longer to live (*n* = 26,945). Overall, nearly half (44.4%) of the sample were 74 years or older, 49.5% were female and 62.5% were married or had a partner. There were no significant differences between those with a longer or shorter prognosis in terms of age, sex, marital status or living arrangement (stdiff < 0.2 in all cases; Table 1).

**Table 1:** Demographic characteristics of palliative home care clients in Ontario, stratified by prognosis

Variable	Overall study population ***N*** = 74,964	Prognosis ≥ 6 months (***n*** = 26,945)	Prognosis < 6 months (***n*** = 48,019)	Standardized difference
	Column % (n)			
**Age group**				
18-44	2.9 (2193)	3.6 (958)	2.6 (1235)	−0.06
45-64	25.9 (19,397)	28.2 (7602)	24.6 (11,795)	0.08
65-74	26.9 (20,136)	27.7 (7470)	26.4 (12,666)	0.03
74-84	27.7 (20,754)	26.2 (7065)	28.5 (13,689)	−0.05
85+	16.7 (12,482)	14.3 (3849)	18.0 (8633)	−0.10
**Sex**				
Male	51.5 (37,844)	48.5 (13,079)	51.6 (24,765)	−0.06
Female	49.5 (37,120)	51.5 (13,866)	48.4 (23,254)	0.06
**Marital status**				
Never married	5.5 (4119)	6.2 (1681)	5.1 (2438)	0.05
Married/partner	62.5 (46,834)	62.0 (16,693)	62.8 (30,141)	−0.02
Widowed	22.8 (17,116)	21.4 (5773)	23.6 (11,343)	−0.05
Separated/divorced	9.2 (6895)	10.4 (2798)	8.5 (4097)	0.07
**Living arrangement**				
Alone	18.0 (13,475)	19.8 (5323)	17.0 (8152)	0.07
With others	82.0 (61,489)	80.2 (21,622)	83.0 (39,867)	−0.07

Cancer was the most prevalent diagnosis in the overall sample (83.2%), however both circulatory (46.2%) and musculoskeletal (18.5%) diseases were also highly prevalent. Diseases of the eyes, ears, skin as well as congenital diseases all had a prevalence of less than 5% in the sample and are therefore not reported in Table 2. Individuals with a shorter prognosis were more likely to experience higher rates across a number of variables, including having shortness of breath at rest (22.1% vs. 13.4%; stdiff = − 0.23), ADL impairment (57.5% vs. 32.0%; stdiff = − 0.53), and moderate to severe symptoms of fatigue (75.3% vs. 59.5%; stdiff = − 0.34; Table 2).

**Table 2:** Physical, psychosocial and spiritual characteristics among palliative home care clients by prognosis

Variable	Overall study population (N = 74,964)	Prognosis ≥6 months (n = 26,945)	Prognosis < 6 months (n = 48,019)	Standardized difference
	Column % (n)			
**Disease Diagnoses**^**a**^				
Cancer	83.2 (62,394)	82.9 (22,326)	83.4 (40,068)	−0.01
Circulatory	46.2 (34,614)	45.8 (12,332)	46.4 (22,282)	−0.01
Musculoskeletal	18.5 (13,861)	19.3 (5208)	18.0 (8653)	0.03
Diabetes	18.3 (13,691)	18.1 (4874)	18.4 (8817)	− 0.01
Respiratory	17.8 (13,344)	17.7 (4760)	17.9 (8584)	−0.01
Nervous/mental disorders	17.6 (13,155)	18.6 (5005)	17.0 (8150)	0.04
Metabolic/endocrine	13.9 (10,382)	14.3 (3865)	13.6 (6517)	0.02
Digestive	12.2 (9237)	11.8 (3174)	12.6 (6063)	− 0.02
**Number of co-morbid chronic conditions**				
0-2	51.8 (38,803)	51.8 (13,955)	51.7 (24,848)	0.02
3+	48.2 (36,161)	48.2 (12,990)	48.3 (23,171)	−0.02
**Activities of Daily Living (ADL-H) Scale**				
Independent/minor supervision (0-1)	51.7 (38,726)	68.0 (18,317)	42.5 (20,409)	0.53
Moderate/severe impairment (2-6)	48.3 (36,238)	32.0 (8628)	57.5 (27,610)	−0.53
**Pain Scale**				
No pain/less than daily (0-1)	29.1 (21,801)	33.5 (9035)	26.6 (12,766)	0.15
Daily/severe pain (2-3)	70.9 (53,163)	66.5 (17,910)	73.4 (35,253)	−0.15
**Cognitive Performance Scale (CPS) Scale**				
No/mild impairment (0-1)	73.8 (55,316)	80.4 (21,650)	70.1 (33,666)	0.24
Moderate/severe impairment (2-6)	26.2 (19,648)	19.7 (5295)	29.9 (14,353)	−0.24
**Depression Rating Scale (DRS)**				
No signs/symptoms of depression (0-2)	87.7 (65,759)	89.3 (24,068)	86.8 (41,691)	0.08
Signs/symptoms of depression (3-14)	12.3 (9205)	10.7 (2877)	13.2 (6328)	−0.08
**Pressure Ulcer Risk Scale**				
Low/mild risk (0-2)	67.6 (50,693)	79.7 (21,486)	60.8 (29,207)	0.42
Moderate/high/very high risk (3-8)	32.4 (24,271)	20.3 (5459)	39.2 (18,812)	−0.42
**Health-related outcomes**				
Fell in last 90 days	25.8 (18,253)	22.0 (5865)	28.0 (12,388)	−0.13
Experiences too much sleep	29.2 (21,899)	19.4 (5225)	34.7 (16,674)	−0.35
Shortness of breath				
Absent	33.0 (24,763)	39.0 (10,518)	29.7 (14,245)	0.20
Absent at rest, present during moderate/day-to-day activities	48.0 (35,979)	47.5 (12,809)	48.3 (23,170)	−0.01
Present at rest	19.0 (14,222)	13.4 (3618)	22.1 (10,604)	−0.23
Moderate to severe fatigue	69.6 (52,184)	59.5 (16,044)	75.3 (36,140)	−0.34
Nausea (present during last 3 days)	33.4 (25,052)	29.4 (7929)	35.7 (17,123)	−0.13
Vomiting (present during last 3 days)	15.3 (11,468)	12.1 (3266)	17.1 (8202)	−0.14
Fluctuating state of consciousness	7.8 (5816)	2.7 (738)	10.7 (5078)	−0.32
Acute change in mental status from person’s usual functioning	10.5 (7815)	4.5 (1212)	13.9 (6603)	−0.33
One or more hospital admissions in last 90 days	46.1 (34,521)	39.4 (10,617)	49.8 (23,904)	−0.19
One or more emergency department visits in last 90 days	25.3 (18,987)	22.6 (6075)	26.9 (12,912)	−0.10
**Psychosocial/Spiritual**				
Has a sense of completion on transfer of financial, legal and other formal responsibilities	70.9 (52,665)	64.4 (17,341)	74.5 (35,324)	−0.22
Individual is accepting of the situation	81.9 (60,849)	81.4 (21,903)	82.2 (38,946)	−0.02
Finds guidance in religion or spirituality	42.8 (31,837)	43.1 (11,610)	42.7 (20,227)	0.01
Expresses a wish to die now	4.5 (3407)	1.7 (469)	6.1 (2938)	−0.23
**Other outcomes**				
Caregiver Risk Evaluation (CaRE)				
Low/moderate risk (1-2)	51.3 (35,994)	59.8 (15,842)	46.2 (20,152)	−0.28
High/very high risk (3-4)	48.7 (34,141)	40.2 (10.627)	53.8 (23,514)	0.28
Person believes performance in physical functioning can still improve	27.2 (19,297)	38.2 (10,193)	20.6 (9104)	0.39
Care professional believes person is capable of improved physical functioning	12.3 (8719)	22.9 (6109)	5.9 (2610)	0.50
Individual is aware of their prognosis	39.4 (27,950)	19.2 (5126)	51.6 (22,824)	−0.72

^a^Disease diagnoses groups are not mutually exclusive

Although some key differences existed, based on prognosis, the two groups were very similar (i.e., stdiff < 0.2 in all cases) across multiple health-related outcomes. For example, the groups experienced similar and high rates of severe/daily pain (73.4% vs. 66.5%), nausea (35.7 vs. 29.4%), hospital admissions (49.8% vs. 39.8%), emergency department visits (26.9% vs. 22.6%), and having at least three co-morbid chronic conditions (48.3% vs. 48.2%). While those with a shorter prognosis were statistically more likely to have a caregiver being at high or very high risk of caregiver burden, a large proportion of caregivers to those with a longer prognosis also fell into this group (53.8% vs. 40.2%; Table 2).

**Fig. 1:**
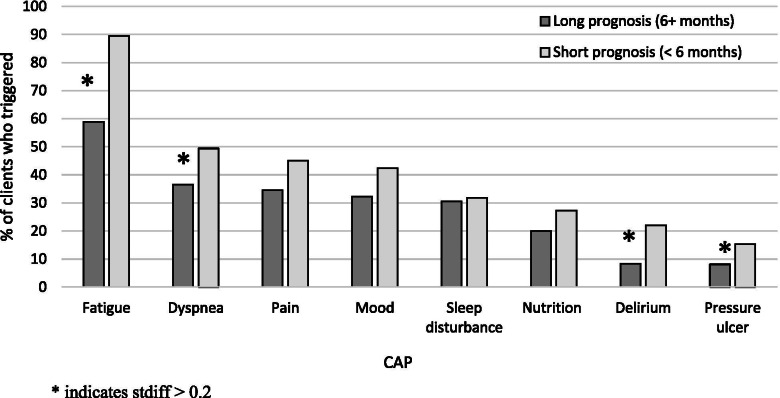
Proportion of individuals that trigger a Clinical Assessment Protocol (CAP) stratified by prognosis

In terms of the CAP triggering rates, we again found that both groups experienced similar rates of triggering across four of the eight (62.5%) CAPs. Both groups triggered the following CAPs at rates that were considered not statistically significant, including pain (45% vs. 34.5%), mood (42.4% vs. 32.3%), sleep disturbance (31.8% vs. 30.6%), and nutrition (27.3% vs. 20%). The exceptions to this were the fatigue CAP, delirium CAP, dyspnea CAP, and pressure ulcer CAP which were all significantly higher in the short prognosis group; Fig. 1).

## Discussion

To date, there are limited studies that touch on the experiences of individual’s receiving PC in their own homes in Ontario. While these studies have utilized interRAI PC data in Ontario, almost all of them were based off of early pilot data, which included a limited sample size and focused on very specific aspects of PC [37–40]. Therefore, this study represents an important step in trying to better understand the overall needs and symptom burden of individuals receiving care in the community, and highlights their complex clinical needs, regardless of prognosis. In the current study, we found that most clients, regardless of prognosis, experienced high rates of severe pain, nausea and caregivers being at a high risk of experiencing burden.

Pain is one of the most common symptoms experienced by palliative home care clients with a life-limiting illness [41]. It is often present with multiple diagnoses and tends to increase in prevalence during the dying process [42]. A recent study by Davidson et al., (2017) found that almost 70% of seriously ill home care patients in Ontario experienced some degree of pain [43]. This is in line with our results as individuals with both a shorter (73%) and longer (67%) prognosis experienced severe/daily pain. Uncontrolled or unaddressed pain can negatively affect not only the individual’s physical functioning, but also their psychosocial well-being and overall quality of life. Additionally, inadequate pain control has been associated with experiencing a “bad death” as most individuals fear dying in pain [41, 44]. It is therefore important to continue to screen for and address all types of pain for any individual with a life-limiting illness, irrespective of prognosis.

The majority of Canadians prefer to “age in place” and remain in their own homes for as long as possible [11]. This means that most individuals with a life-limiting illness would prefer to receive care at home, which oftentimes is provided by an informal caregiver such as a family member or friend. These individuals typically do not have any formal training on how to care for an individual with a life-limiting illness and have to balance not only the physical care for the individual, but also the financial, psychological and emotional aspects of caregiving. This additional strain may increase the likelihood for the caregiver to experience burden [45, 46]. In the current study, we found that 53.8 and 40.2% of caregivers were categorized as being at high or very high risk of experiencing caregiver burden, for both the short and long prognosis groups, respectively. Caregiver burden is correlated with poor mental [47] and physical health [48] and is associated with an increased risk for mortality [49]. Since PC is meant to be holistic in nature and encompass care for the person as well as their family, it is critical to identify caregivers who are at risk of experiencing burden so that appropriate supports can be put in place.

Palliative care is often provided to those with a cancer diagnosis compared to other chronic life-limiting illnesses due to the predictability of decline [4]. Although the PC community is shifting towards providing PC to those with non-cancer diagnoses, in the current study, we still found that approximately 80% of individuals had a cancer diagnosis, regardless of prognosis. However, there were still a number of other life-limiting diagnoses present in this sample, including circulatory diseases (46%), nervous/mental disorders (18%) and metabolic/endocrine disorders (18%). Identifying the appropriate time to initiate PC for those with a non-cancer diagnosis can be challenging due to the complex and unpredictable trajectory of the illness. For most other life-limiting diseases besides cancer, there are no reliable models for identifying prognosis [50]. A recent population-based cohort study for decedents in Ontario examining access to PC by disease trajectory found that those with a terminal illness such as cancer had PC initiated four times earlier than those with other life-limiting illnesses [4]. Prognosis should not be the sole determinant of when PC is initiated, but rather it should be made available based on the needs of the individual and their family [51]. There is no accepted definition of what constitutes early PC, however it has been suggested that PC can begin at the time of diagnosis and can be provided in conjunction with curative treatments [6].

There is a common misconception with some patients and health care professionals that PC is synonymous with end-of-life care. This misconception can lead to individuals not having access to PC until late in their illness trajectory when symptom burden is high [52]. However, there has been a recent shift in the discipline of PC which supports the idea that PC should be available for anyone who could benefit from a palliative approach to their care. This shift has been observed in multiple countries, including Canada, the United Kingdom, the United States, Australia, New Zealand and Belgium [53–58]. The initiation of early PC has been found to improve overall quality of life, reduce rates of depressive symptoms and utilization of health care services [3, 4]. The results of the current study demonstrate that individuals with a longer prognosis experience similar negative health-related outcomes as those with a shorter prognosis. It is important that these individuals continue to be identified early in the illness trajectory so their complex needs are identified, discussed with them and their family and then addressed through a comprehensive and patient-centred care plan.

A potential limitation of the current study is the decision to use everyone’s most recent assessment as we may actually be capturing individuals as they are closer to death. This sample represents a mix of individuals who have been on service for a certain length of time as well as those that were recently referred. We felt it was important to get a cross section of all clients currently on service that was as close to the current situation as possible, as this study serves as a foundation in understanding who receives home PC in Ontario, Canada’s largest province. The majority of individuals (73%) only had one assessment available, therefore even if we used a different assessment (i.e., an individual’s first assessment in the data), we would still be capturing the majority of the same individuals as in the current study. We also completed a sensitivity analysis comparing the results using an individual’s first assessment in the data and found that in the vast majority of circumstances (94%), the difference between proportions when using the first vs. most recent assessment was less than 5%.

Additionally, we recognize that this paper is focused on Ontario, but from an international point of view, this is the only interRAI PC data that is available. Currently, New Zealand is in the process of rolling out the interRAI PC assessment country wide, therefore in the future, our research team plans to do multi-country analysis. Finally, we were unable to examine the person’s primary diagnosis, based on how the diagnoses were captured. However, we were still able to identify the most prevalent diagnoses in the sample, including cancer, circulatory and musculoskeletal diseases. There are also a number of strengths in the study, including the large sample size, which represent all regions in Ontario as the vast majority of regions are using the interRAI PC assessment. The interRAI PC assessment has a large variety of data elements that are not often found in administrative data, which is typically what has been used to look at PC services in the past [59, 60]. Administrative data does not capture information on symptoms such as pain and nausea and no information is captured on caregiver burden, therefore making the interRAI PC an important assessment tool for those receiving PC.

## Conclusion

The needs of home care clients receiving palliative care in Ontario are complex and the results of this research highlight the importance of providing PC to any individual who could benefit from a palliative approach to their care, regardless of diagnosis or prognosis. This information is needed as it allows health care professionals and policy makers to better understand the care needs of individuals being treated in the community. While those with other life-limiting illnesses tend to have a longer prognosis compared to individuals with a cancer diagnosis, these individuals still experience similar issues as those with a shorter prognosis. Therefore, it is vital that anyone living with a life-limiting illness be identified as someone who could benefit from PC as it has the potential to improve their overall quality of life throughout the trajectory of their illness.

## Supplementary Information

**Additional file 1: Table 1**. Description of Clinical Assessment Protocol (CAP) triggering levels. A detailed description of how each individual Clinical Assessment Protocol (CAP) triggering level is defined.

**Additional file 2: Table 2**. Clinical Assessment Protocol (CAP) triggering rates in the overall sample. The triggering rates for each Clinical Assessment Protocol (CAP) across all clients in the sample.

## Data Availability

The data are not publicly available, but they are directly available to interRAI fellows and their staff and students. Other researchers can access the data from the Canadian Institute for Health Information for researchers who meet the criteria for access to confidential data. These data represent third party data that are not owned nor collected by the study authors. A data request form can be found here: https://www.cihi.ca/en/access-data-and-reports/make-a-data-request
