# Supplementary material for: Prognosis does not change the landscape: palliative home care clients experience high rates of pain and nausea, regardless of prognosis
Source: BMC Palliat Care. 2021 Oct 20;20:165. doi: 10.1186/s12904-021-00851-x (PMC8527809; doi:10.1186/s12904-021-00851-x)
Supplement: Supplementary file 1 — Additional file 1: Table 1. Description of Clinical Assessment Protocol (CAP) triggering levels. A detailed description of how each individual Clinical Assessment Protocol (CAP) triggering level is defined. [file 12904_2021_851_MOESM1_ESM.docx]

Additional Table 1: Description of Clinical Assessment Protocol (CAP) triggering levels

| **CAP Name** | **Description** | **Triggering Rules** |
| --- | --- | --- |
| Dyspnea | To determine the cause of the dyspnea and address it to the extent possible | 1. Triggered—moderate or worse dyspnea 2. Not triggered |
| Delirium | Identify and treat underlying cause(s) of delirium to the extent possible | 1. Triggered when behaviours are different from normal functioning (e.g., easily distracted, acute change in mental status) 2. Not triggered |
| Fatigue | Describe the severity of fatigue and determine to degree to which it is a burden to the person | 1. Triggered with severe fatigue 2. Triggered by those at risk of severe fatigue 3. Not triggered |
| Mood | Identify and address any immediate threats to the person’s well-being posed by depression or anxiety. | 1. Triggered with multiple mood symptoms 2. Triggered with single mood symptom 3. Not triggered |
| Nutrition | Ensure person and caregiver understand the unique issues related to nutrition in palliative care and to reduce anxiety about not eating | 1. Triggered with low body mass index (BMI; 20 or lower) but no recent weight loss 2. Triggered with low BMI (20 or lower) and recent weight loss 3. Not triggered |
| Pain | Identify and treat underlying reason(s) for pain and to optimize ability to be comfortable at all times | 1. Triggered at high priority 2. Triggered at medium priority 3. Not triggered |
| Pressure ulcer | Prevent the development of pressure ulcers or prevent an increase in size of current ulcer | 1. Triggered as likely to improve 2. Triggered as difficult to improve 3. Not triggered |
| Sleep disturbance | Identify and determine underlying causes in order to try and maximize person’s comfort and function | 1. Triggered with high potential to improve 2. Triggered with moderate potential to improve 3. Not triggered |
