# Supplementary material for: Prognosis does not change the landscape: palliative home care clients experience high rates of pain and nausea, regardless of prognosis
Source: BMC Palliat Care. 2021 Oct 20;20:165. doi: 10.1186/s12904-021-00851-x (PMC8527809; doi:10.1186/s12904-021-00851-x)
Supplement: Supplementary file 2 — Additional file 2: Table 2. Clinical Assessment Protocol (CAP) triggering rates in the overall sample. The triggering rates for each Clinical Assessment Protocol (CAP) across all clients in the sample. [file 12904_2021_851_MOESM2_ESM.docx]

| **CAP** | **Not triggered** | **Trigger level 1** | **Trigger level 2** |
| --- | --- | --- | --- |
|  | % (n) | | |
| Dyspnea | 55.2 (41,413) | 44.8 (33,551) |  |
| Delirium | 82.9 (61,670) | 17.1 (12,678) |  |
| Fatigue | 21.6 (16,069) | 43.2 (32,075) | 35.2 (26,167) |
| Mood | 61.3 (45,371P) | 18.9 (14,031) | 19.9 (14,759) |
| Nutrition | 75.5 (48,918) | 8.6 (5,592) | 15.9 (10,277) |
| Pain | 58.8 (43,371) | 20.5 (15,144) | 20.7 (15,268) |
| Pressure ulcer | 87.4 (61,938) | 9.2 (6,528) | 3.4 (2,411) |
| Sleep disturbance | 68.7 (51,031) | 11.6 (8,591) | 19.8 (14,759) |

Additional Table 2: Clinical Assessment Protocol (CAP) triggering rates in the overall sample
